# Exogenous Amino Acids Are Essential for Interleukin-7 Induced CD8 T Cell Gowth

**DOI:** 10.1371/journal.pone.0033998

**Published:** 2012-04-17

**Authors:** Claire Pearson, Ana Silva, Benedict Seddon

**Affiliations:** Division of Immune Cell Biology, MRC National Institute for Medical Research, The Ridgeway, Mill Hill, London, United Kingdom; Virgen Macarena University Hospital, Spain

## Abstract

IL-7 signalling is important in regulating both survival and cellular size (growth) of T cells. While glucose metabolism has previously been implicated in the mechanism of IL-7 induced survival and growth, the role of amino acids has not before been reported. Here, we show IL-7 dependent T cell survival does not require either exogenous glucose or amino acids. In contrast, maintenance of cell size and IL-7 induced growth were specifically dependent on amino acids. Furthermore, cellular amino acid uptake was implicated in the mechanism of IL-7 induced growth. Analysis of IL-7 regulated gene expression revealed that neutral and cationic amino acid transporters were specific transcriptional targets of IL-7 signalling. In contrast, none of the four glucose transporters expressed in T cells were modulated. Taken together, these data reveal for the first time the central importance of amino acid homeostasis for IL-7 regulated T cell growth.

## Introduction

The cytokine IL-7 is essential for normal T cell homeostasis. Both thymopoesis [1], [2] and survival of peripheral naïve T cells [3], [4], [5] are dependent on IL-7 signalling. Induction of IL-7R signalling by IL-7 results in the activation of a number of pathways leading to diverse biological outcomes. Dimerisation of IL-7R**α** and **γ**c results in activation of a classical Jak-Stat signalling pathway, mediated by Jaks 1 and 3, and activation of nuclear factor Stat5 [6]. Stat5 is thought to regulate T cell survival by induction of anti-apoptotic factors such as Bcl-2 [7], [8], [9], [10] and cell cycle processes through regulation of cyclins such as Cyclin D1 [11].

IL-7 also regulates the maintenance of T cell size and cellular metabolism. IL-7 induced growth in cellular size is sensitive to PI3kinase and mTOR inhibitors, suggesting that IL-7 signalling via a PI3kinase, Akt and mTOR dependent pathway is involved [12]. Further studies demonstrate that activation of PI3K pathway by IL-7R is in fact a late signalling event dependent on new gene transcription induced by STAT5 activation, rather than by direct PI3K activation downstream of IL-7R [13]. Nutrient transporters have also been specifically implicated in IL-7 induced T cell growth, and specifically those that transport glucose. IL-7 induces increased uptake of glucose by T cells and the facultative glucose transporter, Glut1, is specifically up-regulated by IL-7 signalling [13]. In conditions of limiting glucose *in vitro*, T cell survival is impaired [13]. However, although one would anticipate that cell growth should depend on new protein synthesis, it is unknown whether IL-7 regulates amino acid transport or indeed whether amino acids are required for either IL-7 dependent survival and/or cell growth. In the present study, we compared the requirement for glucose and amino acids for IL-7 dependent survival and growth of T cells. While T cell survival in response to IL-7 stimulation occurred independently of either nutrients, IL-7 induced growth of cells was entirely dependent on the presence of amino acids in culture medium. Furthermore, analysis of IL-7 regulated gene transcription revealed that amino acid transporter expression is a specific target of IL-7 signalling.

## Results

### Neither glucose nor amino acids are required for IL-7 induced survival of naïve CD8 T cells

**Figure 1:**
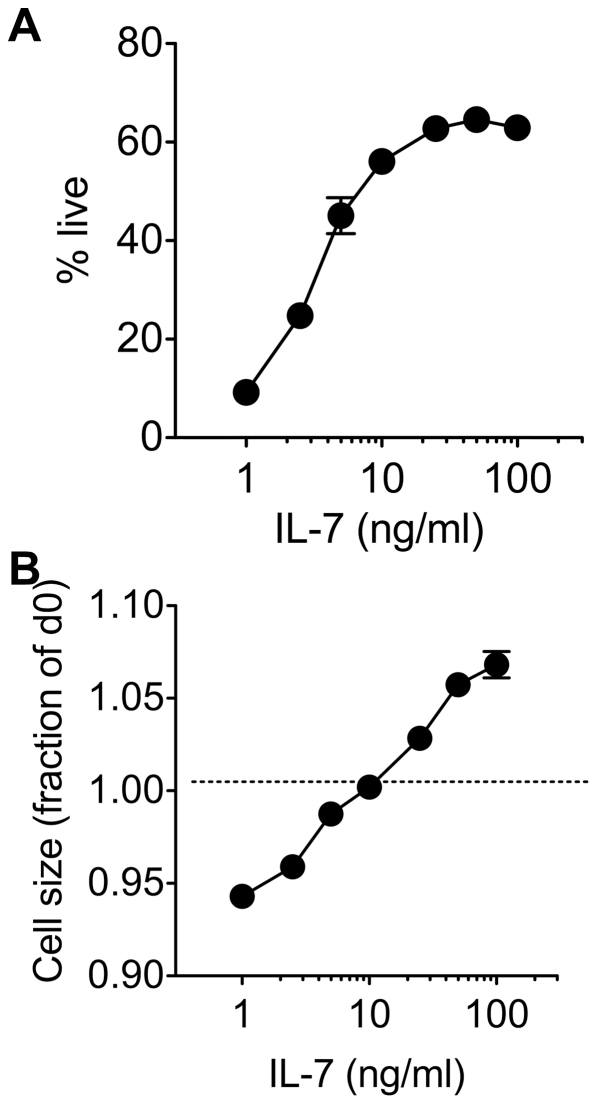
IL-7 mediated growth and survival is dose dependent in vitro. CD8 T cells were enriched from C57Bl6/J donors and cultured with a range of IL-7 concentrations. After 72 h, cells were stained for CD8 expression and with 7AAD and viability and cell size determined by flow cytometry. (A) Graph shows viability of CD8^+^ cells in cultures with different IL-7 concentrations. Viable CD8 T cells were identified as large cells that excluded 7AAD dye. Viability in IL-7 free cultures was <1% at 72 h (data not shown). (B) Graph shows cell size in cultures with different concentrations of IL-7. Data are expressed as size relative to ex vivo CD8 T cell controls. Error bars indicate SD of technical replicates. Results are representative of two or more experiments.

To establish culture conditions compatible with analysing both survival and cellular growth of T cells, we examined the dose dependence of IL-7 for both growth and survival. CD8 T cells from C57Bl6/J wild type (WT) mice were cultured in complete medium at a range of IL-7 concentrations for 72 h, after which cell viability and size was assessed. Increasing concentrations of IL-7 promoted both cell survival and growth of CD8 T cells (Fig. 1). Maximal survival was achieved at a concentration between 25–50 ng/ml (Fig. 1A). Size of individual T cells, as assessed by normalised FSc profile on FACS, was also proportional to IL-7 dose. Maintenance of a cell size comparable to that of ex vivo CD8 T cells required that IL-7 was at least 10 ng/ml, while growth resulting in increased cell size was observed at doses above 10 ng/ml (Fig. 1B). For the studies here, we therefore chose a dose of 50 ng/ml as a compromise between the concentration required for optimal survival (∼25 ng/ml) and the dose at which maximal growth was observed (100 ng/ml).

**Figure 2:**
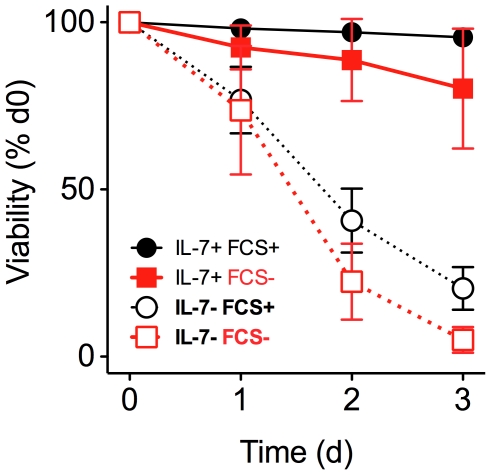
Minimal fetal calf serum requirement for IL-7 dependent survival of CD8^+^ T cells. CD8^+^ T cells were enriched from C57Bl6/J donors and cultured for the times indicated, either alone (open symbols, dashed lines), or in the presence of 50 ng/ml of IL-7 (filled symbols, solid lines) and additionally either in presence (circles) or absence (squares) of foetal calf serum (FCS). Cells were stained with 7AAD and viability amongst CD44^lo^CD8^+^ cells determined by measuring the frequency of 7AAD^−^ live cells by flow cytometry. Percentage of surviving cells was normalized to the percentage of live cells on day 0. Results are pool of three independent experiments. Error bars indicate SD of biological replicates.

**Figure 3:**
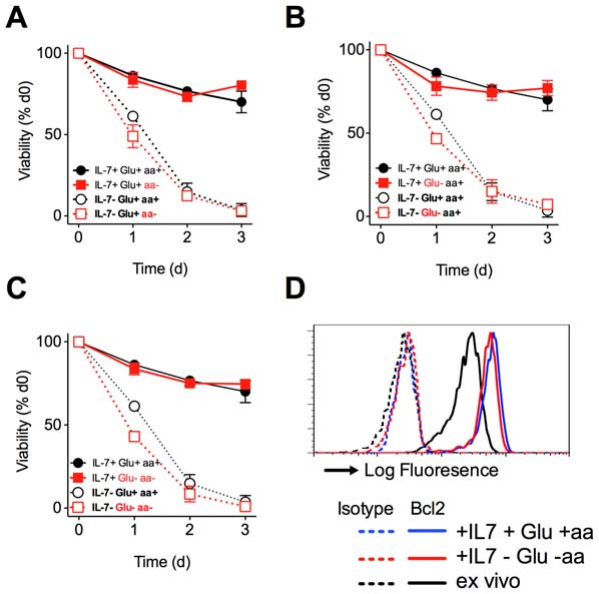
Neither amino acids nor glucose are required for IL-7 dependent survival of naïve CD8 T cells. CD8^+^ T cells were enriched from C57Bl/6J donors and cultured for the indicated time points, either alone (open symbols, dashed lines), or in the presence of 50 ng/ml of IL-7 (filled symbols, solid lines). Cultured cells were stained with 7AAD and frequency of viable 7AAD^−^ cells amongst total CD44^lo^ CD8^+^ T cells determined by flow cytometry. Cells were cultured in RPMI medium containing standard nutrients (circles) or in RPMI specifically lacking (A) amino acids (aa−), (B) glucose (Glu−) or (C) glucose and amino acids (Glu− aa−, squares throughout). (D) CD8^+^ T cells from C57Bl6/J donors were cultured for 24 h in the presence of 50 ng/ml of IL-7, in RPMI either containing standard nutrients (+Glu +aa) or in the absence of glucose and amino acids (−Glu −aa). Cells were stained for Bcl-2 and expression compared with *ex vivo* CD44^lo^CD8^+^ T cells. Histograms show Bcl2 expression by CD44^lo^ CD8^+^ T cells cultured in the presence (blue solid line) and absence (red solid line) of glu and aa, as compared with *ex vivo* CD44^lo^ CD8^+^ T cells (black solid line). Broken lines indicate isotype negative control stainings for each population. (A–C) Error bars indicate SD of technical replicates. Results are representative of three experiments.

To investigate the role of cellular amino acid homeostasis in the function of IL-7 on naïve CD8 T cells, we first examined their requirement for IL-7 dependent T cell survival *in vitro*. CD8 T cells were purified from WT mice and cultured with IL-7 in RPMI medium, or in RPMI medium specifically lacking either amino acids (aa) or glucose (Glu). Survival of naïve CD8 T cells was determined by measuring viability of CD8^+^ CD44^lo^ T cells by 7AAD exclusion. Unless otherwise stated, viability of CD8 T cells refers specifically to these naïve phenotype cells. In aa^+^ Glu^+^ RPMI, IL-7 strongly promoted CD8 T cell survival over the three day culture period that to a large extent did not depend on the presence of fetal calf serum (FCS) (Fig. 2), conventionally an essential component of medium for *in vitro* cultures of cell lines and activated lymphocytes. We therefore omitted the use of FCS from subsequent experiments. IL-7 mediated T cell survival also did not depend on the presence of aa in culture medium (Fig. 3A). In contrast to previous reports [13], we could find no requirement for the presence of Glu in culture medium for IL-7 dependent naïve CD8 T cell survival (Fig. 3B). In confirmation of both the latter results, IL-7 was equally potent at promoting naïve CD8 T cell survival in doubly deficient aa^−^ Glu^−^ RPMI lacking FCS (Fig. 3C). It was possible that memory cells might have distinct requirements for nutrients for their IL-7 dependent survival. To address this, we specifically analysed survival of CD44^hi^ memory phenotype CD8 T cells in the same cultures and found near identical requirements for IL-7 mediated survival as we observed for their naïve counterparts (Fig. S1A–S1C). One mechanism by which IL-7 signaling is thought to promote T cell survival *in vitro* is by the upregulation of Bcl2 expression level (Pearson et al 2011, in press). Examining Bcl2 expression level in cultures revealed that IL-7 dependent upregulation of Bcl2 occurred entirely normally in CD8 T cells cultured either in the presence or absence of aa and Glu (Fig. 3D).

**Figure 4:**
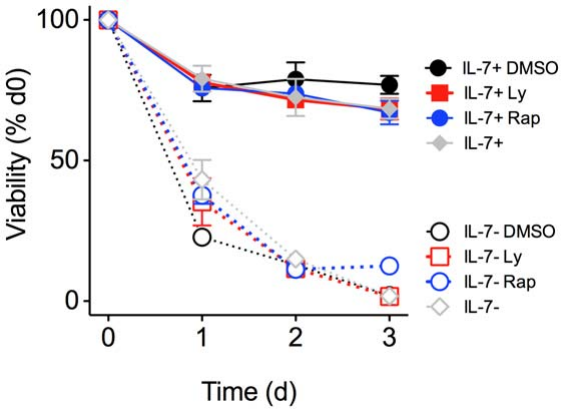
Viability of naïve CD8 T cells is not affected by blockade of PI3K or mTOR. Cells were cultured in RPMI containing standard Glu and aa nutrients and, where indicated, the inhibitors LY294002 (Ly) (10 µM) or rapamycin (Rap) (20 nM), or the vehicle control (DMSO) were added. Percentage of surviving CD44^lo^ CD8^+^ T cells was normalized to the percentage of live CD44^lo^ CD8^+^ T cells on day 0. Error bars indicate SD of technical replicates. Results are representative of three independent experiments.

These data suggested that IL-7 promotes survival of CD8 T cells independently of the need for exogenous nutrients. We therefore sought to reinforce these results by specifically blocking signalling pathways required for cell growth and measuring the ability of IL-7 to promote cell survival. Trophic effects of IL-7 are thought to be downstream of PI3K and mammalian target of rapamycin (mTOR) signalling [12]. Consistent with previous studies [12], neither phosphoinositide-3 kinase (PI3K) inhibitors nor rapamycin, that inhibits mTOR, impaired IL-7 dependent survival of either naïve CD8 T cells (Fig. 4) or CD44^hi^ memory phenotype CD8 T cells in the same cultures (Fig. S1D). Thus, IL-7 is able to promote T cell survival *in vitro* in the complete absence of FCS, aa and Glu, further suggesting that it is the upregulation of Bcl-2 expression levels is the key process by which IL-7 prevents T cell death.

### Exogenous amino acids are essential for IL-7 induced CD8 T cell growth

**Figure 5:**
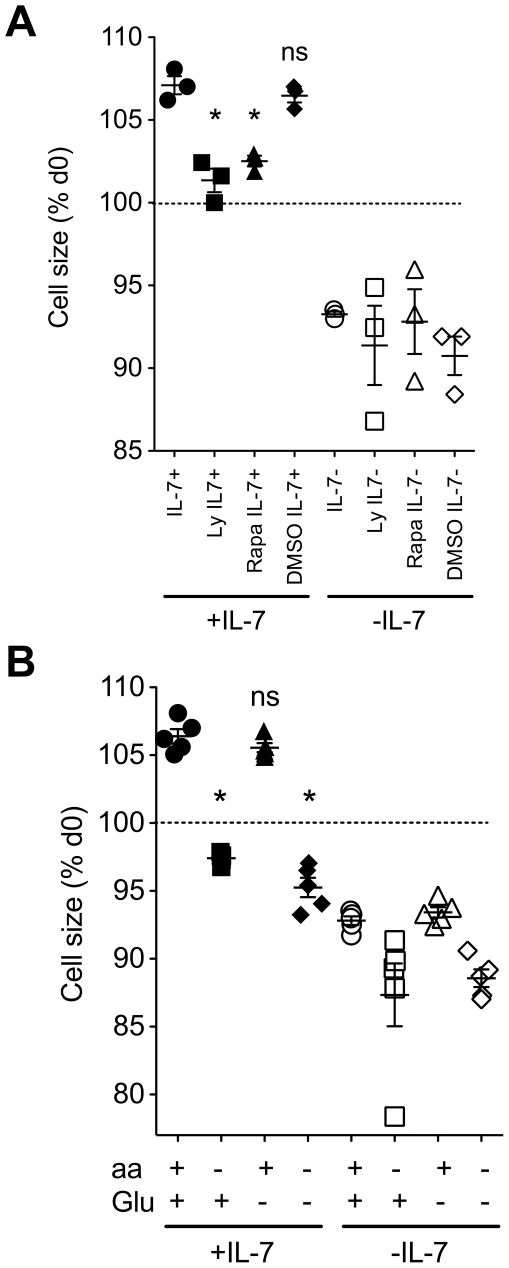
IL-7 induced T cell growth is strictly dependent on exogenous amino acids but not glucose. CD8^+^ T cells were enriched from C57B6/J donors and cultured for 3 days in RPMI containing standard nutrients, either alone (open symbols), or in the presence of 50 ng/ml IL-7 (filled symbols). Cultured cells were stained with 7AAD and size of viable CD44^lo^ CD8^+^ T 7AAD^−^ cells determined by measuring FSc by FACS. (A) Where indicated, the inhibitors LY294002 (Ly, squares) (10 µM), rapamycin (Rap, triangles) (20 nM) or the vehicle control (DMSO, diamonds) were added to cultures. (B) Cells were cultured in RPMI medium containing standard nutrients (circles) or in RPMI specifically lacking amino acids (squares), glucose (triangles) or lacking both glucose and amino acids (diamonds). Cells were cultured in the presence (filled symbols) or absence (empty symbols) of exogenous IL-7 (50 ng/ml). Results are the pool of three or more independent experiments. Each point plotted is the mean of triplicate cultures for a single independent experiment. Error bars indicate SD of biological replicates. * p<0.001; ns, not significant.

As well as regulating T cell survival, IL-7 also plays a role in both maintaining cell size and inducing further T cell growth in size. CD8 T cells from WT mice undergo progressive growth *in vitro* in the presence of IL-7 (Fig. 1B), while in the absence of this cytokine, the remaining viable cells do not maintain their cell size [12]. Consistent with previous studies, IL-7 induced cell growth of naïve CD8 T cells was dependent on the activity of PI3K and mTOR, since pharmacological inhibitors of these pathways specifically inhibited IL-7 induced cell growth (Fig. 5A). To investigate the requirement for aa and Glu in this growth response to IL-7, we cultured CD8 T cells with IL-7 in the presence or absence of exogenous aa or Glu. IL-7 induced identical growth of naïve CD8 T cells when cultured in aa^+^ Glu^+^ and aa^+^Glu^−^ medium, and it appears that Glu in culture medium is not required for IL-7 induced cell growth (Fig. 5B). In contrast, naïve CD8 T cells cultured in medium lacking aa, either in aa^−^Glu^+^ or aa^−^Glu^−^ medium, completely failed to grow in response to IL-7 stimulation, and even shrank in size as compared with the same cells at d0 (Fig. 5B). Interestingly, there was a trend to suggest that naïve CD8 T cells cultured in the absence of IL-7 underwent even greater reduction in cell size specifically in aa deficient media (Fig. 5B). Analysis of growth of CD44^hi^ memory phenotype cells in the same cultures revealed an identical specific requirement for aa for IL-7 induced growth (Fig. S2), as observed for naïve T cells. Hence, exogenous aa are essential for both maintenance of T cell size and IL-7 induced cell growth *in vitro*.

### IL-7 regulates amino acid but not glucose transporter gene expression

**Figure 6:**
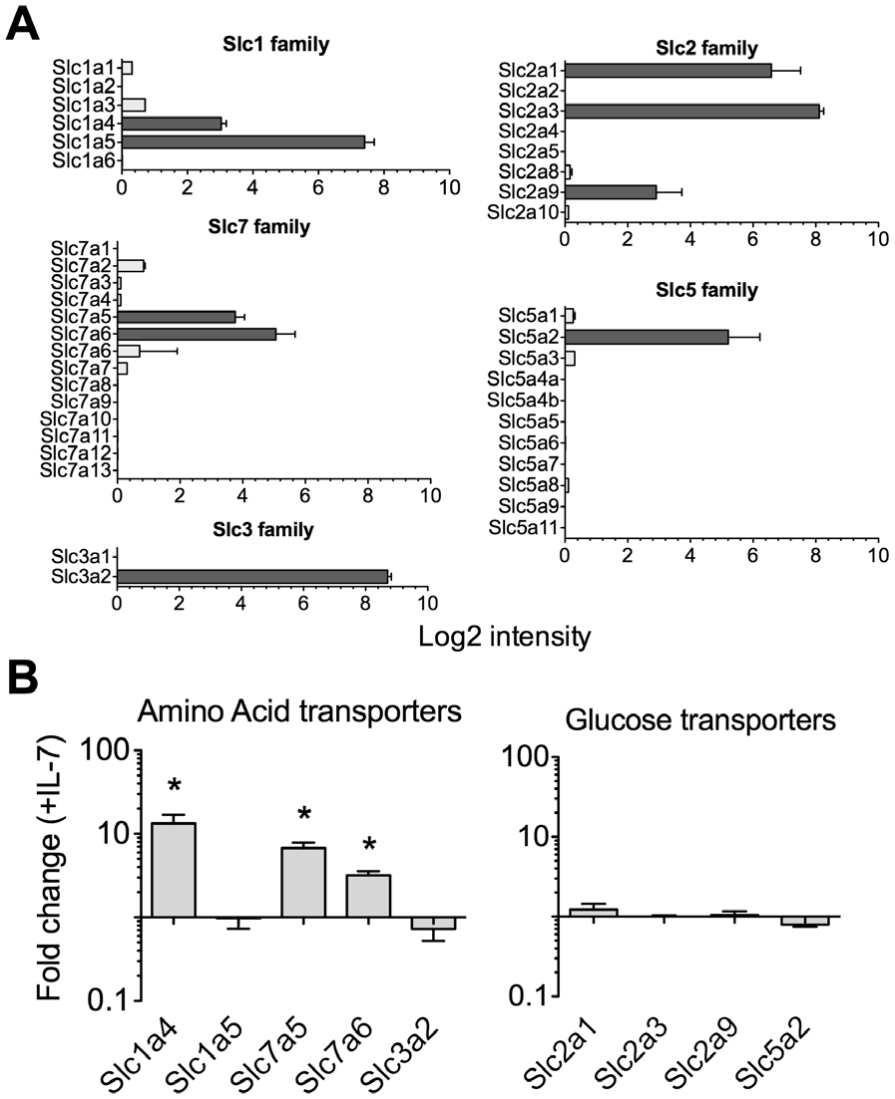
Regulation of amino acid transporter expression by IL-7 signaling. Microarray analysis was performed to compare gene expression in purified CD8^+^ populations of F5 T cells from F5 *Rag1^−/−^* mice *ex vivo* (n = 4) or following culture with 50 ng/ml IL-7 for 24 h (n = 4) (Pearson et, in press). (A) Graphs show relative expression level of indicated genes in F5 T cells *ex vivo*. (B) Graphs indicate fold change in expression of those amino acid and glucose transporters expressed in F5 T cells, following culture with IL-7, as compared with expression in *ex vivo* F5 T cells. Error bars indicate SD of technical replicates. * p<0.05.

Next, we asked whether IL-7 influenced cellular aa and/or glucose homeostasis at the level of specific transporter gene expression. In a previous study, we examined genes regulated by IL-7 signalling (Pearson et al, in press). In this study, microarray analysis was used to identify gene expression changes in F5 TCR transgenic T cells stimulated with IL-7 for 24 h. We used this data set to ask whether IL-7 signalling regulated expression of either glucose or amino acid transporters. The solute carrier (SLC) family comprises a large number of membrane transporter proteins divided into different families. The SLC2 (14 member genes) and SLC5 (12 member genes) families are the facilitative GLUT transporters and sodium glucose co-transporters respectively. SLC1 (7 member genes) and SLC7 (14 member genes) families are high affinity glutamate and neutral amino acid transporters and the cationic amino acid transporter families respectively. The SLC7 family includes the glycoprotein associated light subunit that forms heterodimeric transporter complexes with SLC3A2, the 4F2 cell surface antigen heavy chain. Analysis of gene expression data revealed four amino acid transporter proteins expressed in F5 T cells: Slc1a4, Slc1a5, Slc7a5 and Slc7a6 (Fig. 6A). Similarly, expression of four glucose transporters was detected: Slc2a1, Slc2a3, Slc2a9 and Slc5a2 (Fig. 6A). Significantly, this pattern of expression of SLC1, SLC2, SLC3, SLC5 and SLC7 families was virtually identical to that recorded in the Immunological Genome Project (http://www.immgen.org/index_content.html) for peripheral CD8 naïve T cells, with the exception of Slc7a6, whose expression was not detected in F5 T cells. Comparing gene expression between *ex vivo* and IL-7 stimulated F5 T cells revealed that transcription of three of the four amino acid transporters expressed in F5 T cells was substantially increased by IL-7 signalling (Fig. 6B). In contrast, none of the glucose transporter genes were affected by IL-7 signalling (Fig. 6B).

**Figure 7:**
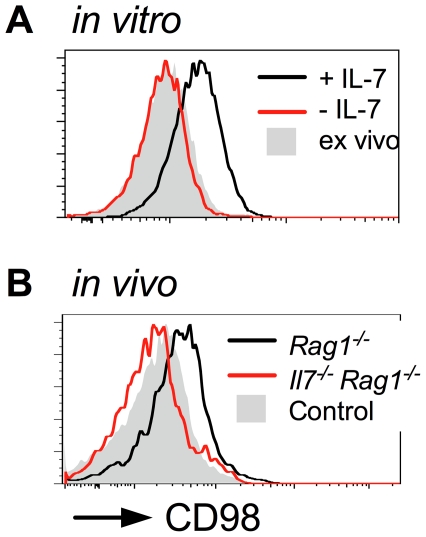
IL-7 regulates CD98 expression levels in vitro and in vivo. CD8^+^ T cells from C57Bl6/J mice were either cultured *in vitro* in the presence or absence of IL-7 for 24 h, or transferred to either *Rag1* ^−/−^ or *Il7* ^−/−^ *Rag1* ^−/−^ hosts. Upper histograms show CD98 expression by CD44^lo^CD8^+^ cells cultured for 24 h either alone (red line) or in the presence of 50 ng/ml IL-7 (black line) as compared with *ex vivo* control CD44^lo^CD8^+^ T cells (grey fill). Lower histograms show CD98 expression in CD44^lo^CD8^+^ cells transferred to *Rag1^−/−^* (black line) or *Il7^−/−^ Rag1^−/−^* hosts (red line) for 3 days, as compared with *ex vivo* control CD44^lo^CD8^+^ T cells (grey fill). Results are representative of three independent experiments.

Finally, we examined CD98 protein expression in WT CD8 T cells by flow cytometry. CD98 is the heterodimeric amino acid transporter complex of *Slc7a5* and *Slc3a2* gene products that form the large neutral amino acid transporter (LAT1). In vitro, stimulation of WT CD8 T cells with IL-7 resulted in a substantial increase in CD98 levels as compared with cells from IL-7 free cultures or ex vivo CD8 T cells (Fig. 7A). To test whether CD98 expression could also be regulated in vivo, CD8 T cells from WT mice were adoptively transferred to lymphopenic *Rag1* ^−/−^ hosts and CD98 expression levels assessed three days later. CD98 expression was elevated on CD8 T cells from *Rag1* ^−/−^ hosts as compared with control T cells from WT mice to a similar extent as observed by T cells simulated with IL-7 in vitro (Fig. 7B). Induction was found to be IL-7 dependent, since the same cells transferred to *Il7^−/−^ Rag1* ^−/−^ hosts failed in upregulate CD98 (Fig. 7B).

## Discussion

The role of IL-7 in regulating metabolism by influencing glycolysis is well recognised. IL-7 signalling maintains glucose uptake and glycolysis, possibly by upregulation of Pim1 [13] that is implicated in increased glycolysis. Whether IL-7 signalling affects abundance or function of glucose transporters appears to depend upon circumstance. We found no evidence of transcriptional regulation of any of the glucose transporters expressed in F5 T cells. However, there is evidence that Glut1 is regulated by post-transcriptional control of protein trafficking to the cell surface [13]. One notable difference between our data and previous studies [13], is that we found glucose was not required for IL-7 mediated T cell survival in vitro. However, as previously reported [12], we found that IL-7 mediated survival and upregulation of Bcl2 expression did not depend on PI3K or mTOR activity. Since IL-7 induced glucose uptake is dependent on PI3K or mTOR pathways [13], the observations that CD8 T cell survival did not require either exogenous glucose or PI3K/mTOR pathways are consistent with one another. Together, these data strongly suggest that IL-7 dependent T cell survival does not absolutely depend upon increased glucose uptake stimulated by IL-7 signaling. The view that IL-7 promotes T cell survival independently of its influence on glucose uptake is also supported by other studies showing that conditional loss of IL-7R expression by T cells *in vivo* results in their rapid loss in vivo [3], [14], without affecting their uptake of glucose [14].

Remarkably, we found that IL-7 could promote T cell survival in vitro in a minimal RPMI medium lacking both exogenous glucose and amino acids. Significantly, IL-7 signaling still resulted in upregulation of Bcl2, which is likely to be a key anti-apoptotic mediator. However, since naïve CD8 T cells are resting quiescent cells, it can be speculated that their requirements for such nutrients is minimal given their relative inactivity, and a combination of cellular reserves of amino acids and turnover of proteins may be sufficient to meet requirements for new protein synthesis, at least in the short term. This appeared to be the case over the 72 h of the experiments reported here. The dose of IL-7 used in the present study was chosen on the basis that it both promoted substantial and measurable cell growth but was below the dose required for maximal survival of CD8 T cells at 72 h. Future studies could determine whether the requirement for exogenous amino acids and glucose for T cell survival is different at lower levels of IL-7 stimulation.

In contrast to the requirements for IL-7 mediated CD8 T cell survival, we found that IL-7 induced growth was absolutely dependent on exogenous amino acids but not glucose. While CD8 T cell survival was maintained by IL-7, in media specifically lacking amino acids, T cells failed to grow in response to IL-7 stimulation. We also found evidence that IL-7 signalling was specifically regulating cellular homeostasis of amino acids. Of the four amino acid transporters expressed by T cells, we found that IL-7 stimulation specifically upregulated gene expression of three, suggesting that IL-7 signalling is tuning amino acid homeostasis by regulating expression of these transporters. While a passive nutritional requirement for amino acid uptake with respect to protein synthesis for cell growth is logical, our data suggest that amino acids also play an active role in regulating growth. Exogenous amino acids are known to inhibit autophagy [15], that would divert amino acids from cell proteins to essential processes. However, amino acids are also found to induce S6K1 activation that is dependent on mTOR activity [16], suggesting that they may also play an active signalling role in inducing T cell growth in response to IL-7 in cooperation with mTOR.

The potential influence of metabolic pathways by exogenous nutrients such as amino acids has important implications for immune responses. Blocking mTOR activity *in vivo* has been shown to enhance memory cell development [17]. Nutrient deprivation, either in draining lymph nodes or at the site of ongoing infections where there is high metabolic demand from large numbers of infiltrating cells, may therefore affect the fate of effector T cells, by tuning S6K1 activity to promote generation of resting memory cells, that are metabolically less demanding than active effectors that might otherwise undergo apoptosis. Th17 cell differentiation has also been shown to be affected by abundance of amino acids, since amino acid deprivation blocks Th17 differentiation [18], and aromatic amino acids are precursors of aryl hydrocarbon receptor ligands that also promote Th17 induction [19]. IL-7 promotes Th17 responses in vivo [20], therefore regulation of amino acid transport could play a role in this process. In conclusion, we present clear evidence that amino acid uptake is crucial for IL-7 induced growth by normal T cells, and predict that perturbation of amino acid uptake will have a profound affect on normal T cell function.

## Materials and Methods

### Mouse strains used in this study

C57Bl6/J and F5 *Rag1−/−*, *Rag1* ^−/−^, *Il7^−/−^ Rag1^−/−^* mice were bred in a conventional colony free of pathogens at the NIMR, London. Animal experiments were performed according to institutional guidelines and United Kingdom Home Office regulations.

### Flow cytometric analysis

Flow cytometry was carried out using lymph node or spleen cells. Cell concentrations were determined using a Scharfe Instruments Casy Counter (Scharfe System, Reutlingen, Germany). Cells were incubated with saturating concentrations of antibodies in 200 µl PBS-bovine serum albumin (0.1%)-azide (1 mM) for 30 mins at 4°C followed by two washes in PBS-bovine serum albumin-azide. Monoclonal antibodies used in this study were as follows: CD8a (eBioscience), APC-TCRb (H57-597; eBioscience), FITC-TCRb (BD Biosciences), FITC-CD44 (IM7; eBioscience), APC-CD4 (eBioscience), APC-CD98 Alexafluor647 (Serotec). Cell viability was measured by FACS, using forward scatter signal as a measure of cell size and exclusion of 7-aminoactinomycin D (7AAD, Sigma) by live cells. Four colour cytometric staining was analysed on a FACSCalibur (Becton Dickinson, San Jose, CA). Data was analysed using Flowjo software v8.1 (Tree Star, Ashland, OR). PE-Bcl-2 (BD Biosciences, PharMingen) staining of samples fixed with IC fix buffer (eBioscience), was carried out according to the manufacturer's instructions.

### Cell isolation and culture

For *in vitro* culture experiments, CD8^+^ T cell were purified >90% by magnetic-activated cell sorting (MACS) using anti CD8a microbeads and LS columns (Miltenyi Biotec, Bergisch Gladbach, Germany) following the manufacturer's instructions. CD8^+^ T cells were cultured in 96 well plates at 1×10^6^ cells/ml at 37°C with 5% CO2 in RPMI 1640 medium (Sigma-Aldrich, St Louis, MO and manufactured in house, NIMR), supplemented with 2-mercaptoethanol and antibiotics (all Sigma-Aldrich). Both standard and bespoke RPMI media lacking aa and/or Glu were made by the NIMR Media Preparation Team. Where used, IL-7 (Peprotech, Rocky Hill, NJ) was supplemented at 50 ng/ml. Where used, LY294002 (Sigma) was used at 10 µM, and rapamycin (Calbiochem) was used at 20 nM.

### Statistics

Statistical significance of changes in cell size (Figs. 5A, 5B and S2) were tested by One way Anova test with Dunnetts multiple comparison test. * p<0.001; **ns**, not significant. Statistical significance of differences in gene expression level in IL-7 stimulated CD8 T cells (Fig. 6B) were tested by non-parametric Mann Witney test. * p<0.05.

## Supporting Information

Figure S1 **Neither amino acids, glucose, PI3K nor mTOR are required for IL-7 dependent survival of memory CD8 T cells.** CD8^+^ T cells were enriched from C57Bl/6J donors and cultured for the indicated time points, either alone (open symbols, dashed lines), or in the presence of 50 ng/ml of IL-7 (filled symbols, solid lines). Cultured cells were stained with 7AAD and frequency of viable 7AAD^−^ cells amongst total CD44^hi^ CD8^+^ memory phenotype T cells determined by flow cytometry. (A–C) Graphs show cell viability of cells cultured in RPMI medium containing standard nutrients (circles) or in RPMI specifically lacking (A) amino acids (aa−), (B) glucose (Glu−) or (C) glucose and amino acids (Glu− aa−, squares throughout). (D) Cells were cultured in RPMI containing standard Glu and aa nutrients and, where indicated, the inhibitors LY294002 (Ly) (10 µM) or rapamycin (Rap) (20 nM), or the vehicle control (DMSO) were added. Percentage of surviving CD44^hi^ CD8^+^ T cells was normalized to the percentage of live CD44^hi^ CD8^+^ T cells on day 0. Error bars indicate SD of biological replicates. Results are pool of three independent experiments.
(TIF)

Figure S2 **IL-7 induced T cell growth is strictly dependent on exogenous amino acids but not glucose.** CD8^+^ T cells were enriched from C57B6/J donors and cultured for 3 days in RPMI containing standard nutrients, either alone (open symbols), or in the presence of 50 ng/ml IL-7 (filled symbols). Cultured cells were stained with 7AAD and size of viable CD44^hi^ CD8^+^ T 7AAD^−^ cells determined by measuring FSc by FACS. Cells were cultured in RPMI medium containing standard nutrients (circles) or in RPMI specifically lacking amino acids (squares), glucose (triangles) or lacking both glucose and amino acids (diamonds). Cells were cultured in the presence (filled symbols) or absence (empty symbols) of exogenous IL-7 (50 ng/ml). Results are the pool of three or more independent experiments. Error bars indicate SD of biological replicates. * p<0.001; ns, not significant.
(TIF)
