# Correction: Exogenous Amino Acids Are Essential for Interleukin-7 Induced CD8 T Cell Growth

**DOI:** 10.1371/annotation/ee9c3e82-855f-42e0-8e57-83981bc1ba16

**Published:** 2012-08-02

**Authors:** Claire Pearson, Ana Silva, Benedict Seddon

Due to an error introduced during the production process, the word "Growth" was misspelled in the article title. The correct title is: Exogenous Amino Acids Are Essential for Interleukin-7 Induced CD8 T Cell Growth. The correct citation is: Pearson C, Silva A, Seddon B (2012) Exogenous Amino Acids Are Essential for Interleukin-7 Induced CD8 T Cell Growth. PLoS ONE 7(4): e33998. doi:10.1371/journal.pone.0033998

