# Supplementary figures and images for: Exogenous Amino Acids Are Essential for Interleukin-7 Induced CD8 T Cell Gowth
Source: PLoS One. 2012 Apr 17;7(4):e33998. doi: 10.1371/journal.pone.0033998 (PMC3328464; doi:10.1371/journal.pone.0033998)

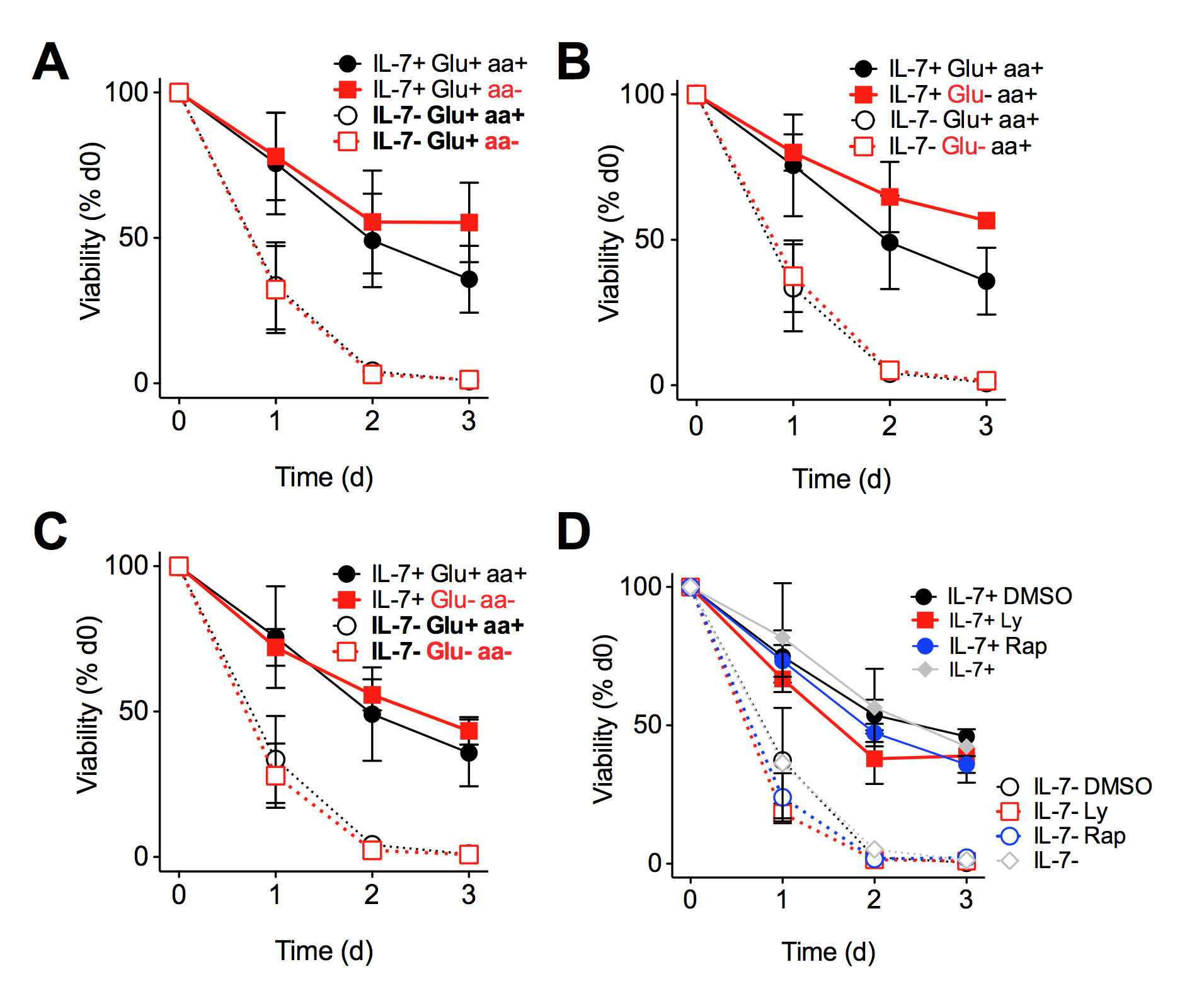

Supplement: Figure S1 — Neither amino acids, glucose, PI3K nor mTOR are required for IL-7 dependent survival of memory CD8 T cells. CD8+ T cells were enriched from C57Bl/6J donors and cultured for the indicated time points, either alone (open symbols, dashed lines), or in the presence of 50 ng/ml of IL-7 (filled symbols, solid lines). Cultured cells were stained with 7AAD and frequency of viable 7AAD− cells amongst total CD44hi CD8+ memory phenotype T cells determined by flow cytometry. (A–C) Graphs show cell viability of cells cultured in RPMI medium containing standard nutrients (circles) or in RPMI specifically lacking (A) amino acids (aa−), (B) glucose (Glu−) or (C) glucose and amino acids (Glu− aa−, squares throughout). (D) Cells were cultured in RPMI containing standard Glu and aa nutrients and, where indicated, the inhibitors LY294002 (Ly) (10 µM) or rapamycin (Rap) (20 nM), or the vehicle control (DMSO) were added. Percentage of surviving CD44hi CD8+ T cells was normalized to the percentage of live CD44hi CD8+ T cells on day 0. Error bars indicate SD of biological replicates. Results are pool of three independent experiments. (TIF) [file pone.0033998.s001.tif]
